# Improved inference of site-specific positive selection under a generalized parametric codon model when there are multinucleotide mutations and multiple nonsynonymous rates

**DOI:** 10.1186/s12862-018-1326-7

**Published:** 2019-01-14

**Authors:** Katherine A. Dunn, Toby Kenney, Hong Gu, Joseph P. Bielawski

**Affiliations:** 1 Department of Biology, Dalhousie University, Halifax, Nova Scotia B3H 4J1 Canada; 2 Department of Mathematics & Statistics, Dalhousie University, Halifax, Nova Scotia B3H 4J1 Canada; 3 Centre Comparative Genomics and Evolutionary Bioinformatics (CGEB) at Dalhousie University, Halifax, Canada

**Keywords:** Codon model, Positive selection, Protein evolution, Multiple nucleotide mutations, Multiple nonsynonymous rates, M-series models, G-series models, Likelihood ratio test, False positives, Model misspecification, Codon frequencies

## Abstract

**Background:**

An excess of nonsynonymous substitutions, over neutrality, is considered evidence of positive Darwinian selection. Inference for proteins often relies on estimation of the nonsynonymous to synonymous ratio (*ω* = *d*_N_/*d*_S_) within a codon model. However, to ease computational difficulties, *ω* is typically estimated assuming an idealized substitution process where (*i*) all nonsynonymous substitutions have the same rate (regardless of impact on organism fitness) and (*ii*) instantaneous double and triple (DT) nucleotide mutations have zero probability (despite evidence that they can occur). It follows that estimates of *ω* represent an imperfect summary of the intensity of selection, and that tests based on the *ω* > 1 threshold could be negatively impacted.

**Results:**

We developed a general-purpose parametric (GPP) modelling framework for codons. This novel approach allows specification of all possible instantaneous codon substitutions, including multiple nonsynonymous rates (MNRs) and instantaneous DT nucleotide changes. Existing codon models are specified as special cases of the GPP model. We use GPP models to implement likelihood ratio tests for *ω* > 1 that accommodate MNRs and DT mutations. Through both simulation and real data analysis, we find that failure to model MNRs and DT mutations reduces power in some cases and inflates false positives in others. False positives under traditional M2a and M8 models were very sensitive to DT changes. This was exacerbated by the choice of frequency parameterization (GY vs. MG), with rates sometimes > 90% under MG. By including MNRs and DT mutations, accuracy and power was greatly improved under the GPP framework. However, we also find that over-parameterized models can perform less well, and this can contribute to degraded performance of LRTs.

**Conclusions:**

We suggest GPP models should be used alongside traditional codon models. Further, all codon models should be deployed within an experimental design that includes (*i*) assessing robustness to model assumptions, and (*ii*) investigation of non-standard behaviour of MLEs. As the goal of every analysis is to avoid false conclusions, more work is needed on model selection methods that consider both the increase in fit engendered by a model parameter and the degree to which that parameter is affected by un-modelled evolutionary processes.

**Electronic supplementary material:**

The online version of this article (10.1186/s12862-018-1326-7) contains supplementary material, which is available to authorized users.

## Background

Markovian models of codon evolution have been extensively developed and tested over the last decade, largely due to their value in investigations of functional divergence at the molecular level (see Anisimova and Liberles [1] for a recent review). Unlike an amino acid model, the rate of evolution prior to selection at the level of the protein (i.e., the rate of synonymous codon substitution, or *d*_S_) can be readily estimated under a model of codon substitution. Comparing that rate to the rate of evolution after the effect of selection on the protein (i.e., the rate of nonsynonymous codon substitutions, or *d*_N_) leads to an easily interpretable index of natural selection pressure. Specifically, the ratio *ω* = *d*_N_/*d*_S_ is estimated from a dataset and interpreted in terms of purifying selection (*ω* < 1), neutral evolution (*ω* = 1), or positive selection (*ω* > 1). Codon models used in this way can be divided into two very broad groups based on their treatment of how physiochemical properties of amino acids might impact the probability of a nonsynonymous substitution. One group of models assumes a single instantaneous rate for all amino acid exchanges. This leads to a single selective regime (i.e., one *ω*) for all nonsynonymous substitutions, regardless of how radical or conservative a change in amino acid physiochemical property. We follow Delport et al. [2] in referring to these as *single-nonsynonymous rate* (SNR) models (see Table 1 for definitions of all the model-related acronyms used in this study). The other group of models attempt to relax the SNR restriction by permitting *multiple-nonsynonymous rates* (MNR). Interestingly, SNR models are much more widely used in studies of protein functional divergence despite well-known variability in amino acid replacement rates, as inferred from large protein sequence databases [3–5].

**Table 1:** Descriptions of the model-related acronyms

**Acronym**	**Description**
DT	Indicates that a model allows simultaneous double (D) and triple (T) nucleotide changes between codons
G0	A GPP codon model employing a single *ω* parameter
G1a^X^	A GPP codon model with the same discrete mixture of two *ω* parameters as model M1a; the total number of free parameters in the model is given by X, and varies depending on how DT and exchangeabilities are modeled
G2a^X^	A GPP codon model with the same discrete mixture of three *ω* parameters as model M2a; the total number of free parameters in the model is given by X, and varies depending on how DT and exchangeabilities are modeled
GPP	General-Purpose Parametric (GPP) modelling framework for codons
GTR	General Time Reversible (GTR) model for single nucleotide changes
GY	The codon modelling framework of Goldman and Yang [26] where the transition probability is proportional to the target codon frequency
M0	A codon model employing a single *ω* parameter as implemented in PAML [54]
M1a	A codon model employing a constrained discrete mixture of two *ω* parameters [45]
M2a	A codon model employing a constrained discrete mixture of three *ω* parameters [45]
M3	A codon model employing an unconstrained discrete mixture of *k* independent *ω* parameters [6]
M8	A codon model employing a discretized *β* distribution to model among site variability in *ω* [6]
MEP	Mixed Empirical and Parametric (MEP) models combine empirical estimates of exchangeabilities with so-called mechanistic parameters of codon evolution
MG	The codon modelling framework of Muse and Gaut [47] where the transition probability is proportional to the target nucleotide frequency
MNR	A class of models allowing Multiple Nonsynonymous Rates (MNR) of exchangeability between codons
PCP	Physiochemical-Constrained Parametric (PCP) models parameterize the influence of physiochemical constraints on nonsynonymous changeability
REV	A fully reversible codon model described by a 61×61 matrix *Q*, where all codon exchangeabilities are independent parameters of the model.
SNR	A class of models allowing only a Single Nonsynonymous Rates (SNR) of exchangeability between codons

The primary reason for employing SNR models is computational convenience. In addition to needing only a single *ω* parameter, substitutions between codons having two or more nucleotide differences are often assigned zero probability. By employing both restrictions, the number of parameters in the codon rate matrix is reduced from thousands to just a few. For example, in addition to *ω*, a typical formulation might only require parameters for the transition/transversion ratio (*κ*) and the equilibrium codon frequency of the *i*^th^ codon (*π*_*i*_). Such simplification facilitates the extension SNR models to permit variation in selection regimes among sites (e.g., [6, 7]), branches [8], or both (e.g., [9, 10]) while keeping model complexity low enough for single-gene datasets. Simulation studies indicate that extending SNR models in this way substantially increases power to detect adaptive molecular evolution (e.g., [7, 9, 11]), and experimental assessment of the results of SNR models has validated their utility in a wide variety of real datasets (e.g., [12–15]).

One strategy for model improvement is to increase mechanistic realism while avoiding over parameterization [16]. Thus, modelling variability in amino acid exchangeabilities through MNR codon models should improve inferences about functional divergence [2, 17, 18]. However, given the size and complexity of the codon rate matrix, this is a challenging task and a variety of strategies have been explored. Here, we divide those strategies into three categories: (*i*) mixed empirical and parametric (MEP) models; (*ii*) physiochemical-constrained parametric (PCP) models and (*iii*) general-purpose parametric (GPP) models. Below we provide a brief review of those models implemented for the purpose of making inference about the process of molecular evolution. Note that Schneider et al. [19] were the first to construct a codon model having heterogeneous amino acid exchangeabilities. Because the purpose of their model was to aid the process of alignment it will not be considered further.

MEP models combine empirical estimates of exchangeabilities with so-called mechanistic parameters of codon evolution (e.g., *ω*, *κ*, and *π*_*i*_). Doron-Faigenboim and Pupko [17] chose to integrate existing empirical amino acid exchangeability matrices with such mechanistic parameters. In this situation, nonsynonymous exchangeabilities between codons are set equal to amino-acid exchangeabilities (189 parameters) previously derived from large sets of amino acid sequences. Kosiol et al. [18] used a massive dataset to estimate the first fully empirical codon model (1830 codon exchangeability parameters) and then combined those with mechanistic parameters for codon evolution. De Maio et al. [20] subsequently reduced that model’s complexity while maintaining comparable performance. The empirical matrices in these studies represent very broad averages of the propensity for amino acid change. Miyazawa [21] and Zoller and Schneider [22] developed different methods to tailor the information contained within an empirical exchangeability matrix to a specific dataset. The advantage of all these MEP approaches is that they separate the DNA level evolutionary process from the effect of selection acting on the protein. However, the *ω* parameter of MEP models no longer has the same interpretation as other codon models because database-derived exchangeability values reflect a broadly averaged effect of selection, and these influence the data-specific estimates of selection pressure derived from the *ω* parameter [18, 23].

Building upon the well-known relationship between substitution rates and the physiochemical differences of amino acids (e.g., Clark [24]; Grantham [25]), the PCP models explicitly parameterize the influence of physiochemical constraints on nonsynonymous changeability. Goldman and Yang [26] and Yang, Nielsen and Hasegawa [27] employed explicit mathematical functions to model the relationship between the *ω* parameter and physiochemical properties, and Yang [28] allowed the influence of the physiochemical property to vary among sites. Sainudiin et al. [29] and Wong et al. [30] implemented models that partition nonsynonymous changes into a small number of categories according to a pre-defined physiochemical property. As the purpose of those models was to test if certain physiochemical properties might be subject to natural selection, their parameterization is focused on comparing the rate of property-altering substitutions to the rate of property-conserving substitutions. Conant and Stadler [31] accounted for multiple amino acid properties by modelling exchangeabilities between nonsynonymous codons as a linear combination of five pre-specified measures of physiochemical property. The advantage of these PCP approaches is that they permit investigation of explicit relationships between physiochemical properties and selection pressure while seeming to avoid over parameterization of the codon model. However, the PCP approach requires strong assumptions about the relative importance of different properties, and they are not well suited to assessing the fit of alternative property scales (which are often non-independent). The space of possible physiological constraints is vast, and any given set of constraints neglects the potential importance of unique structural factors.

The GPP models are fundamentally different from the MEP and PCP models in two ways: (*i*) they do not impose empirically estimated exchangeabilities on individual datasets, nor do they require the nonsynonymous substitution rate to depend on a pre-specified physiochemical property, and (*ii*) they seek to identify the best approximation of a fully-reversible (REV) codon model (a 61 × 61 *Q* matrix that fully determines the dynamics of the codon substitution process) for a given sequence alignment. The REV codon model is attractive because it is a way of relaxing the unrealistic restriction that all amino acid changes have a single instantaneous rate. The cost, however, is an independent parameter for the rate of exchangeability between every unique pair of amino acids, which is far too parameter-rich for an individual gene. Hence, the analytical objective of the GPP approach is to explain a set of data using as few MNR parameters as possible. Delport et al. [2] developed a promising model search-strategy based on a genetic algorithm (GA). The GA is employed to search for the best assignment of amino acid pairs to a set of exchangeability parameters, where the number of such model parameters is also estimated from the data. Zaheri, Dib and Salamin [32] developed a novel analytical framework whereby the full instantaneous rate matrix for codons (3721 elements) can be estimated from just 19 model parameters. The full codon matrix is obtained by using Kronecker product to combine three 4 × 4 nucleotide matrices specified for each position of the codon. Both approaches appear to capture important aspects of real protein-coding sequence evolution, but via very different strategies. However, the parameters of the 4 × 4 nucleotide matrices employed by Zaheri, Dib and Salamin [32] are not defined with respect to an explicit process of codon evolution, which limits their use for testing of codon-level evolutionary processes.

Double and triple (DT) nucleotide substitutions between codons are biologically possible [33–35] as successive changes on a rapid time scale (e.g., promoted by compensatory pressures [36]), via mechanistic processes such as error-prone polymerase activity [37] or during the process of DNA break repair (e.g., Sakofsky et al. [38]). Although such rates are several orders of magnitude lower than single nucleotide substitutions between codons [39–41], models that permit DT changes yield significant improvements in their fit to real data, suggesting that they could be an important addition to codon models. Models allowing DT changes between codons include those of Doron-Faigenboim and Pupko [17], Kosiol, Holmes and Goldman [18], De Maio et al. [20], Miyazawa [21], Zoller and Schneider [22], Zaheri, Dib and Salamin [32], Venkat et al. [42] and Jones et al. [43].

De Maio et al. [20] suggest that some widely used models for *ω* heterogeneity could yield high false positive rates when applied to data where both MNRs and DT codon changes occur. The recent study by Venkat et al. [42] found that double changes alone can induce high false positive rates when branch-site codon models are used in branch-specific tests for positive selection. The MNR models of Delport et al. [2] and Zaheri, Dib and Salamin [32], as currently implemented, do not yet allow among-codon heterogeneity in *ω*. SNR models developed by Jones et al. [43] and Venkat et al. [42] are site-heterogeneous and permit multiple changes between codons, but do not permit MNRs or a general time reversible (GTR) nucleotide model. Because the GTR model has the maximum number of exchangeability (6) and frequency parameters (4) compatible with time-reversibility, it should help avoid the negative effect of model violations for the DNA-level substitution process [7, 44]. Here we introduce a novel pair of GPP models that benefit from (*i*) permitting DT codon changes, (*ii*) a full GTR nucleotide model, (*iii*) MNRs via heterogeneous amino acid exchangeabilities, and (*iv*) estimation of *ω* that is not confounded by average amino acid exchangeabilities estimated from a large database of proteins*.* These new models, referred to as G1a and G2a, use a discrete *ω* distribution similar to those used in the SNR models M1a and M2a [6, 45]. The *ω* distributions similar to M1a and M2a were chosen because the likelihood ratio test (LRT) derived from them appears to have reasonable power while maintaining some robustness to model misspecification [46]*.* These GPP models can be extended further so that the instantaneous rate matrix can take any form up to the REV codon model. We use simulation to evaluate testing for sites under positive selection under several different formulations of models G1a and G2a. We conclude by applying these models to a set of transmembrane proteins from *Streptococcus*.

## Methods

### SNR codon models M0, M1a, M2a, M3 and M8

Goldman and Yang [26] and Muse and Gaut [47] independently proposed similar formulations for modelling the Markovian substitution process between sense codons. Here we present the core formulation of Goldman and Yang [26], as it was developed into models that form some LRTs investigated within this study. The instantaneous substitution rate between codon *i* and *j* (*i* ≠ *j*) at a single site within an alignment of protein coding sequences is defined as:

$$ {q}_{ij}=\left\{\begin{array}{c}0,\ \mathrm{i}\mathrm{f}\ \mathrm{i}\ \mathrm{a}\mathrm{nd}\ \mathrm{j}\ \mathrm{differ}\ \mathrm{by}\ \mathrm{more}\ \mathrm{than}\ \mathrm{one}\ \mathrm{nucleotide}\\ {}{\pi}_j,\ \mathrm{i}\mathrm{f}\ \mathrm{i}\ \mathrm{a}\mathrm{nd}\ \mathrm{j}\ \mathrm{differ}\ \mathrm{by}\ \mathrm{a}\ \mathrm{synonymous}\ \mathrm{transversion}\\ {}\kappa {\pi}_j,\ \mathrm{i}\mathrm{f}\ \mathrm{i}\ \mathrm{a}\mathrm{nd}\ \mathrm{j}\ \mathrm{differ}\ \mathrm{by}\ \mathrm{a}\ \mathrm{synonymous}\ \mathrm{transition}\ \\ {}\omega {\pi}_j,\ \mathrm{i}\mathrm{f}\ \mathrm{i}\ \mathrm{a}\mathrm{nd}\ \mathrm{j}\ \mathrm{differ}\ \mathrm{by}\ \mathrm{a}\ \mathrm{nonsynonymous}\ \mathrm{transversion}\\ {}\omega \kappa {\pi}_j,\ \mathrm{i}\mathrm{f}\ \mathrm{i}\ \mathrm{a}\mathrm{nd}\ \mathrm{j}\ \mathrm{differ}\ \mathrm{by}\ \mathrm{a}\ \mathrm{nonsynonymous}\ \mathrm{transition}\end{array}\right. $$

where the matrix *Q* specifies a continuous-time, stationary, time-reversible Markov process. Parameters *π*_*j*_, *κ* and *ω* specify the stationary frequencies of codon *j*, the transitions to transversion rate ratio, and the nonsynonymous to synonymous rate ratio, respectively. Because this formulation models all nonsynonymous changes using a single *ω* parameter, this is an example of a SNR model. The transition probability matrix *P*(*t*) is related to matrix *Q* by *P*(*t*) = *e*^*Qt*^, thereby giving the probabilities for state changes over a branch of length *t*. The likelihood of a codon site for a given phylogenetic tree and branch lengths can then be calculated using the pruning algorithm of Felsenstein [48]. The above formulation is widely referred to as model M0, and it assumes that the intensity of natural selection (as captured by parameter *ω*) is the same for all sites in the codon sequence alignment. Model M0 was extended to a series of models that permit the *ω* parameter to vary among sites [6], which includes the models known as M1a, M2a, M3 and M8. Hereafter, the family of codon models derived from M0 that permit the *ω* parameter to vary among sites will be referred as “M-series” models. All members of the M-series family are SNR models.

Models M1a and M2a [45] are widely used as the basis of an LRT for positive selection, and for empirical Bayes identification of positively selected sites within a multi-species alignment [49]. These models employ a restricted form of the *ω* distribution that, although highly idealized, leads to desirable properties for the LRT [11, 46]. Model M1a (a.k.a. nearly neutral) is a discrete mixture of two classes of sites: strictly neutral sites with *ω*_1_ = 1, and sites subject to purifying selection with *ω*_0_ estimated from the data but constrained to take a value < 1. The mixture weights for these classes of sites (*p*_0_ and *p*_1_) also are estimated from the data. Model M2a extends model M1a by adding a third class of sites for positive selection (*ω*_+_ > 1). As these models are nested they serve as the basis of a LRT for sites evolving by positive selection.

Model M3 employs an unconstrained discrete distribution for *ω* [6]. In this model, sites are assumed to belong to *k* discrete classes, each having a parameter for selection (*ω*_*i*_) and a proportion of sites (*p*_*i*_) within the gene. An LRT of M3 against M0 (a special case of M3 where *k* = 1 and all sites have just a single *ω*) constitutes a test for variable selection intensity among sites [11]. In this study we use the LRT of M0 versus M3_*k* = 2_ to pre-screen the real datasets and thereby ensure each contains signal for among-site variation in the intensity of natural selection.

Model M8 uses a flexible *β* distribution to permit *ω* to vary among sites within the interval (0,1) and an extra discrete category that can allow *ω*_+_ > 1 [6]. For computational convenience the *β* distribution is divided into 10 bins. An LRT for positive selection is obtained by comparing a restricted form of M8 (*ω*_+_ = 1, fixed) to an alternative form of M8 (*ω*_+_ ≥ 1, estimated). In both models the mixture weights for the *β* distribution (*p*_0_) and *ω*_+_ (*p*_+_) are estimated from the data. This LRT represents a popular alternative to M1a and M2a as a test for sites evolving by positive selection.

### GPP codon models G1a and G2a

We developed GPP codon models that employ the same discrete distributions for *ω* as employed by M1a and M2a, but without requiring that any other simplifying assumptions be imposed on the data (e.g., SNRs, zero probability for DT changes, and restrictions on the GTR). These models are hereafter referred to as G1a and G2a. Like M1a, model G1a assumes that data evolve under one of two discrete selective regimes: purifying selection and strict neutral evolution. Model G2a extends this by adding a class of sites evolving under positive selection. The restrictions, as well as the notation, are the same for the *ω* parameters (*ω*_0_ < 1, *ω*_1_ = 1, and *ω*_+_ > 1) and mixture weights (*p*_0_, *p*_1_ and *p*_+_).

G1a and G2a are derived from a simple GPP codon model that includes the current models such as Goldman and Yang [26] and Muse and Gaut [47] as special cases. We refer to the basic form of this model, which has only a single class of sites, as G0. The GPP model exploits the fact that a time-reversible process is expressible as the product of a matrix of exchangeability parameters (*R*) and the steady state frequencies (*π*), and uses a logarithm link function to link the non-zero off-diagonal elements of the 61 × 61 instantaneous codon matrix, *Q = Rπ*, to a linear model format (see online Additional file 1 for details). We assume *R* is symmetric, and the instantaneous rates can be written as *q*_*ij*_ = *π*_*j*_*r*_*ij*_, where *π*_*j*_is the equilibrium frequency of the *j*^th^ codon, and the parameter *r*_*ij*_ determines the exchangeability between codons. In G0 the matrix of exchangeability parameters, *R*, is determined by a set of model parameters, *β*_0_, …, *β*_*n*_. For each *β*_*k*_ there is a corresponding matrix *X*^(*k*)^, and the value of *r*_*ij*_ for *i* ≠ *j* is determined by *log*(*r*_*ij*_) = ∑_*k*_*β*_*k*_(*X*^(*k*)^)_*ij*_. The diagonal elements of *R* are set such that rows of *Q* sum to 0. The first model parameter, *β*_0_, is a scaling factor set so that the branch lengths can be interpreted as the expected numbers of substitutions per codon sites, and the other parameters *β*_1_, …, *β*_*n*_ are intended to represent different mechanisms of the evolutionary process. This framework allows specification of all possible instantaneous codon substitutions, and any restrictions on the process are special cases of the general model where the instantaneous rate is set to zero (e.g., prohibition of codon substitutions involving DT nucleotide changes is a special case of the general model).

As the familiar SNR codon model M0 [26] is a special case of G0, it serves as a convenient way to illustrate how a GPP model is specified. M0 can be expressed within the GPP framework as follows:

$$ {q}_{ij}=\left\{\begin{array}{c}0,\ \mathrm{i}\mathrm{f}\ \mathrm{i}\ \mathrm{a}\mathrm{nd}\ \mathrm{j}\ \mathrm{differ}\ \mathrm{by}\ \mathrm{more}\ \mathrm{than}\ \mathrm{one}\ \mathrm{nucleotide}\\ {}{e}^{\beta_0}{\pi}_j,\ \mathrm{i}\mathrm{f}\ \mathrm{i}\ \mathrm{a}\mathrm{nd}\ \mathrm{j}\ \mathrm{differ}\ \mathrm{by}\ \mathrm{a}\ \mathrm{synonymous}\ \mathrm{transversion}\\ {}{e}^{\beta_0}{e}^{\beta_1}{\pi}_j,\ \mathrm{i}\mathrm{f}\ \mathrm{i}\ \mathrm{a}\mathrm{nd}\ \mathrm{j}\ \mathrm{differ}\ \mathrm{by}\ \mathrm{a}\ \mathrm{synonymous}\ \mathrm{transition}\ \\ {}{e}^{\beta_0}{e}^{\beta_2}{\pi}_j,\ \mathrm{i}\mathrm{f}\ \mathrm{i}\ \mathrm{a}\mathrm{nd}\ \mathrm{j}\ \mathrm{differ}\ \mathrm{by}\ \mathrm{a}\ \mathrm{nonsynonymous}\ \mathrm{transversion}\\ {}{e}^{\beta_0}{e}^{\beta_1}{e}^{\beta_2}{\pi}_j,\ \mathrm{i}\mathrm{f}\ \mathrm{i}\ \mathrm{a}\mathrm{nd}\ \mathrm{j}\ \mathrm{differ}\ \mathrm{by}\ \mathrm{a}\ \mathrm{nonsynonymous}\ \mathrm{transition}\end{array}\right. $$

where ${e}^{\beta_0}$ is the required matrix scale factor, ${e}^{\beta_1}$ is equivalent to the transition/transversion rate ratio (*κ*), and ${e}^{\beta_2}$ is equivalent to the nonsynonymous/synonymous rate ratio (*ω*). Transitions are indicated by a matrix *X*^(1)^ whose entries are 1 for all single nucleotide changes between codons that are transitions (and 0 for all other entries). Nonsynonymous changes are indicated by a matrix *X*^(2)^ whose entries are 1 for all single nucleotide changes that yield a change in the encoded amino acid (and 0 for all other entries). Note that the requirement that *q*_*ij*_ = 0 if *i* and *j* differ in more than one nucleotide position is explicitly enforced after applying the link function. By removing this requirement and extending *X*^(1)^ and *X*^(2)^ to include DT changes, we obtain an extension of G0 that permits multiple nucleotide changes between codons.

Model G0 (like model M0) is a SNR model because the nonsynonymous exchangeabilities are all equal. However, nonsynonymous exchangeabilities need not be constrained in this way. Any number of mechanisms for differences in nonsynonymous exchangeabilities can be added to the model through additional *β*_*i*_ parameters. For example, empirical data indicate that differences in hydrophobicity among pairs of amino acids is well known to impact the probability of an amino acid substitution (e.g., Clark [24]; Grantham [25]). Taking hydrophobicity as an example, a matrix of pairwise differences in hydrophobicity between amino acids can be constructed from a given scale (e.g., HI of Monera et al. [50]), and the nonsynonymous transition rate can then be linked to the exponent of the entries in this matrix via ${e}^{\beta_{HI}}$, where *β*_*HI*_ is a fitted parameter in the model. Any such addition to the model yields a process of codon evolution having MNRs. Restrictions on the DNA-level process of evolution also can be relaxed. For example, rather than the single parameter for the transition/transverion rate ratio (*β*_1_, in the above model), each DNA-level exchangeability can be modelled with a separate parameter (*β*_*AC*_, *β*_*CT*_, *β*_*AT*_, *β*_*TG*_, *β*_*CG*_). This leads to a codon model having a GTR process at the DNA level, which has been recommended when testing for positive selection (e.g., Kosakovsky Pond and Frost [7]).

Parameterization of a codon model in terms of *β*_1_, …, *β*_*n*_ means that process-variation among sites can be modelled with different random effects for different model parameters. In this study we develop GPP models motivated by M1a and M2a by using constrained discrete distributions to model among site variation in the nonsynonymous rate (*β*_2_ in the above model). These models (G1a and G2a) extend M1a and M2a by permitting double and triple changes between codons, a full GTR process at the DNA level, and model MNRs via the addition of *β*_1_, …, *β*_*n*_ for different aspects of physiochemical constraints.

### Simulation based assessment of the G-series and M-series models

**Fig. 1:**
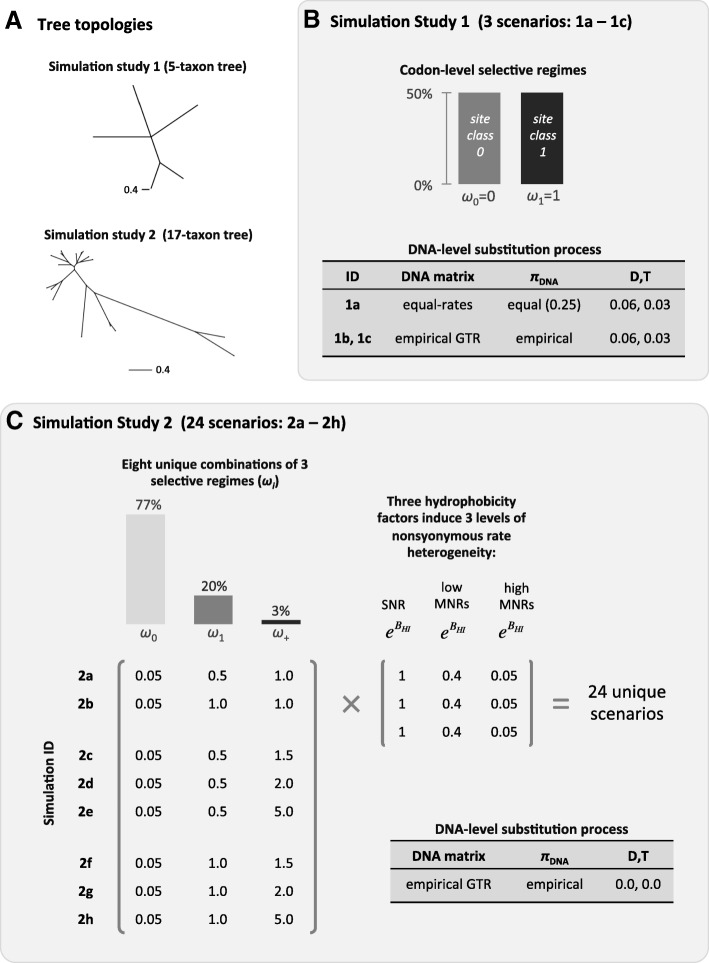
Graphical illustration of the design of Simulation Studies 1 and 2. The overall design is comprised of 32 distinct evolutionary scenarios divided into four distinct Simulation studies focused on different objectives. The details of Simulation studies 1 and 2 are shown in this figure. The details of Simulation Studies 3 and 4 are derived from Studies 1 and 2, and are further explained in the text. All simulation studies were comprised of 100 replicates, each having sequences of 300 codons. All datasets were generated using version 1.2 of the COLD program “www.mathstat.dal.ca/~tkenney/Cold/, https://github.com/tjk23/COLD”. **a** The 5-taxon and 17-taxon tree topologies**.** The 5-taxon tree and branch lengths are the same as those used for simulating sequences in Wong et al. [45]. The 17-taxon tree and branch lengths are the same as those used for simulating sequences in Yang et al. [6]. The scale for the branch lengths gives the mean number of substitutions per codon. **b** Sequence generating process for Simulation Study 1. The purpose of this study is to investigate the impact of DT changes (0.06 and 0.03 respectively) on the false positive rate. The selective regime is based on a strictly neutral model having just two classes of sites; conserved (50% of data) having *ω* = 0 and neutral (50% of data) having *ω* = 1. The scenarios of this study differ in the complexity of the nucleotide substitution process; case 1a is simple (everything equal) and case 1b/1c is complex (unequal GTR exchangeabilities and nucleotide frequencies). The GTR exchangeabilities and nucleotide frequencies for case 1b/1c were obtained from *β*-globin gene sequences. **c** Sequence generating process for Simulation Study 2. This study has 24 scenarios, and covers more complexity than the strictly neutral case of Study 1. Each has a mixture of three selective regimes: a large fraction of strong purifying selection (77%, *ω*_0_ = 0.05), a moderate fraction of sites (22%) with *ω*_1_ = 0.5 or 1.0, and a small fraction evolving with *ω* ≥ 1 (3% *ω*_+_ = 1.0, 1.5, 2.0 or 5.0). MNRs were induced using hydrophobicity factors $\left({e}^{\beta_{HI}}\right)$ of 1.0, 0.4 or 0.05, which were linked to the codon model via the GPP parameter *β*_*HI*_. When ${e}^{\beta_{HI}}=1$ there is no impact on nonsynonymous rates, yielding a SNR codon model. When ${e}^{\beta_{HI}}<1$, codon evolution has MNRs. The nucleotide process had heterogeneous GTR exchangeabilities, and unequal nucleotide frequencies at the three positions of the codon. DT codon changes were not included in Study 2; DT was added to MNRs in Simulation Study 3

Simulation is used to evaluate MLE estimation under the new G-series models and the performance of several LRTs for positive selection (e.g., G1a vs G2a). Our overall design is comprised of 32 distinct evolutionary scenarios (Fig. 1), which serve as the basis for four simulation studies focused on different ways in which model based inference could be impacted. Although the evolutionary details differ between the 32 scenarios, each is comprised of 100 replicate datasets, each having sequences of 300 codons in length.

Simulated datasets were generated using methods implemented in version 1.2 of the COLD program “www.mathstat.dal.ca/~tkenney/Cold/”. COLD is an open source software package available for download from the COLD website “www.mathstat.dal.ca/~tkenney/Cold/”, and from GitHub “https://github.com/tjk23/COLD”. The commands used to generate the sequence data for this study, the relevant Newick tree files, and all multi-sequence alignments that were produced for each of the simulation studies, are available to download from the DRYAD repository for this study [51].

#### Simulation study 1

The purpose of this study is to investigate the impact of DT codon changes on the false positive rate. For this simulation we start with the 5-taxon tree and branch lengths of Wong et al. [45] (Fig. 1a). The generating process for this study is based on a selective regime at the codon level derived from a strictly neutral model of codon evolution (Fig. 1b). In this scenario 50% of the sites are subject to perfect purifying selection (*ω* = 0) and 50% are subject to neutral evolution (*ω* = 1). This scenario is often included in simulation studies as a “benchmark case” for LRTs (e.g., Kosakovski Pond and Frost [7]; Anisimova et al. [11]; Wong et al. [45]; Bao et al. [46]). Here, we extend this benchmark case by adding DT changes between codons, with rates 0.06 and 0.03 respectively. These are in accordance with the notion that their rates are substantially lower than the rate of single nucleotide substitution between codons [39, 40]. To enhance interpretability, we began by setting all GTR exchangeabilities to 1 and specified equal nucleotide frequencies. This scenario is referred to as case 1a (Fig. 1b). We then extended this simulation study in two ways. The first extension was to increase the complexity of the nucleotide-level process by adding unequal GTR exchangeabilities and nucleotide frequencies (from [6]). This extension is referred to as case 1b (Fig. 1b). The next extension was designed to investigate the impact of taxon sampling. Each terminal branch of the 5-taxon tree in Fig. 1a was split by the addition of a second lineage, resulting in a 10-taxon tree. The total length of the new tree (sum of the branches) was set equal to that of the 5-taxon tree, but with the tree length re-distributed evenly among all branches (see online Additional file 2). Simulation over the 10-taxon tree was based on the more complex process of case 1b, and is referred to as case 1c. Each dataset was analysed with M1a and M2a, and variants that permit DT changes, hereafter called G1a^DT^ and G2a^DT^).

#### Simulation study 2

The purpose of this study is to investigate model performance using much more complex scenarios than the strictly neutral case above. The tree and branch lengths are derived from a set of 17 real *β*-globin sequences (Fig. 1a), and thus are the same for all scenarios. This tree has been used widely in previous simulation studies (e.g., [6, 11]). This study is comprised of 24 distinct scenarios (Fig. 1c). Each scenario is based on a mixture of sites having three distinct selective regimes. All scenarios have a large fraction of sites (77%) dominated by purifying selection (*ω*_0_ = 0.05). A moderate fraction of sites (20%) assumed to evolve under moderate purifying selection (*ω*_1_ = 0.5) or neutrality (*ω*_1_ = 1.0). A small fraction of sites (3%) evolve with *ω* ≥ 1 (*ω*_+_ = 1.0, 1.5, 2.0 or 5.0). In addition we also employ heterogeneous GTR exchangeabilities, and unequal nucleotide frequencies at the three positions of the codon, as estimated from a set of real *β*-globin sequences. Lastly, we cover a range of nonsynonymous rate heterogeneity by specifying hydrophobicity factors $\left({e}^{\beta_{HI}}\right)$ of 1.0, 0.4 or 0.05. The hydrophobicity index of Monera et al. [50] was re-scaled by a factor of 100, so that it takes values in the interval [− 1,1], and the absolute value of the difference between the hydrophobicity of amino acids was computed for all pairs of amino acids. The matrix of these scores (online Additional file 3) was linked to the nonsynonymous substitution rate via a parameter in the GPP generating process (*β*_*HI*_). When *β*_*HI*_ = 0, the matrix of hydrophobicity scores will have no impact on nonsynonymous rates, yielding a SNR codon model (${e}^{\beta_{HI}}=1\Big)$. When ${e}^{\beta_{HI}}=0.4\ \mathrm{and}\ 0.05$, the process of codon evolution has MNRs, with ${e}^{\beta_{HI}}=0.05$ yielding an extremely biased MNR model. As our primary interest is the effect of MNRs, we do not include DT codon changes in this study. Note that hydrophobicity is used for convenience to induce MNRs here; any property scale can be similarly used within this GPP framework. Figure 1c indicates the relationship between the different scenarios in this study.

Each scenario was analysed with three different pairs of models. The first was the pair of SNR models M1a and M2a. This pair represents an under-fit modelling scenario. The second pair was G1a^*x*^ and G2a^*x*^, which represent GPP models having perfect fit to the generating process. The superscript of *x* represents the number of mechanistic model parameters required for a perfect fit to a given scenario. The third pair of models was G1a^13^ and G2a^13^. In addition to the branch lengths, and each model’s parameters for the *ω* distribution, these models have *x* = 13 additional parameters. The 13 additional parameters account for DT changes (2 parameters), 6 amino acid properties (polarity, volume, hydropathy, isoelectric point, polar requirement & composition), and GTR exchangeabilities (5 free parameters). Models G1a^13^ and G2a^13^ are used here to represent an over-fit modelling scenario.

#### Simulation study 3

The purpose of this study is to extend Study 2 by adding simultaneous DT nucleotide changes between codons. To minimize the computational burden, the impact of DT nucleotide changes was explored in a selected subset of six scenarios covered in Simulation Study 2. Specifically, we chose three different distributions for *ω* (see 2b, 2d, 2g in Fig. 1)*,* and applied two hydrophobicity factors to each one. The hydrophobicity factors $\left({e}^{\beta_{HI}}\right)$ were 1.0 (yielding an SNR model) and 0.05 (yielding a highly variable MNR model). One *ω* distribution excluded positive selection (77% *ω* = 0.05 and 23% *ω* = 1.0). The other two *ω* distributions included positive selection (77% *ω* = 0.05; 20% *ω* = 0.50; 3% *ω* = 2.0, and 77% *ω* = 0.05; 20% *ω* = 1.0; 3% *ω* = 2.0). As in Study 2, the tree, branch lengths, GTR parameters and codon frequencies were derived from a set of real *β*-globin sequences. Also like Study 2, we used an under-fit model pair (M1a and M2a), a perfectly fit model pair (G1a^*x*^ and G2a^*x*^), and an over-fit model (G1a^13^ and G2a^13^).

#### Simulation study 4

The purpose of this study was to investigate the impact of alternative model formulations on false positive rates for the M-series LRTs. Users of M-series models have many choices for how to (*i*) model the distribution of *ω* variability among sites, and (*ii*) parameterize codon frequencies within the model. A comprehensive assessment of alternative *ω* distributions is beyond the scope of this study. For this reason we chose to assess the LRT for positive selection that compares M8_*ω* + =1_ with M8_*ω*+ > 1_ because it is a popular alternative, and because M8 is based on a discretized *β* distribution. There are two fundamentally different approaches to parameterize codon frequencies. One of them emphasizes the context of the nucleotide change within the complete codon, and employs the equilibrium frequency of the target codon (*π*_*j*_) to model transition probabilities ([26], hereafter denoted GY). The other emphasizes the independence of the process of mutation among sites, and employs the equilibrium frequency of the target nucleotide (*j*) at a single position (*k*) averaged over all codons (*π*_*j*_^*k*^) to model transition probabilities among codons ([47], hereafter denoted MG). Both approaches employ estimates of four nucleotide frequencies at each position of the codon (denoted F3 × 4), and thus each requires 9 free parameters. Despite having similar instantaneous rate matrices, these two Markov processes have different properties when codon frequencies are uneven (e.g., [52]). To investigate the effect of both kinds of modelling choices (*ω* distribution and codon frequencies), we applied both frequency parameterizations (*π*_*j*_ vs. *π*_*j*_^*k*^) to the LRT of M1a vs. M2a and to the LRT of M8_*ω* + =1_ with M8_*ω*+ > 1_. This comparison yields four LRTs per simulation scenario, and because we were interested in false positive rates we applied those four LRTs to all nine null scenarios of Simulations Studies 1–3 (SNR: 1a, 1b, 1c, 2a, 2b & 3a; MNR: 2a, 2b & 3a). In these we covered M-series false positive rates for DT changes, MNRs, and the combination of DT changes and MNRs.

### Real data analyses

We analyzed a set of 24 *Streptococcus* transmembrane proteins. The data are derived from a previous phylogenomic analysis of *Streptococcus* genomes [53]. The homologous gene clusters identified in that study were filtered for clusters of transmembrane proteins with ≥4 unique sequences. The sequence alignments for these gene clusters range from 4 to 19 lineages, and included pathogens and their non-pathogenic relatives. The data were then pre-screened with a LRT for among-codon heterogeneity in *ω* ([6]: M0 vs M3). Three genes had no significant evidence for heterogeneity in *ω* according to this LRT and were excluded from subsequent analyses. The remaining 21 genes were tested using a pair of models that are (presumably) under-fit with respect to DT changes and MNRs (M1a and M2a), and a pair that can be considered mechanistically over-fit for at least some of their parameters (G1a^13^ and G2a^13^). As we do not know the true generating process for these data, we cannot analyze them using a perfectly fit model pair.

### Likelihood calculations and likelihood ratio tests

The values of the model parameters, including branch lengths, were estimated from the data via maximum likelihood. The only exception was the equilibrium frequencies, which were obtained from the empirical codon frequencies within each dataset. The SNR codon models M0, M3, M1a, M2a and M8 were fit to the data as implemented in the codeml program of the PAML package [54]. Fitting the G-series models described above was made possible by an efficient Hessian calculation for phylogenetic likelihood [55], and the GPP modelling framework implemented in version 1.2 of the COLD program “www.mathstat.dal.ca/~tkenney/Cold/”. Model M1a differs from M2a only in the parameters of the *ω* distribution. As these models are nested, and differ by two free parameters, the log likelihood statistic (2Δ) should be approximately *χ*^2^ distributed with 2 degrees of freedom. However, the alternative model (M2a) is related to the null model (M1a) by fixing one of its mixture weights on the boundary (*p*_+_ = 0). This means that the LRT statistic ${\chi}_2^2$ is not the correct distribution; however, we use it here because it is expected to be conservative in many scenarios. The GPP models used in this study employ the same *ω* distributions, and their LRTs are carried out in the same way.

The method for calculation of phylogenetic likelihood under a GPP model is fully described in Kenney and Gu [55]. The implementation of the unique hessian likelihood calculation, and the optimization routines employed to fit the GPP models to sequence data, are distributed via the COLD package as open source software “www.mathstat.dal.ca/~tkenney/Cold/, https://github.com/tjk23/COLD”. COLD uses a variety of metrics to monitor convergence, but COLD’s main convergence test is whether the expected improvement from the next step is less than 1e-10. To deal with some difficult cases, COLD will also claim convergence if the moving average of either expected or actual improvement is less than 1e-5, and will signal if the program has failed to make progress for a long time. Problematic cases of optimization are indicated when either (*i*) COLD fails to converge within the maximum number of iterations, or (*ii*) the likelihood of an alternative model is lower than the null (indicating convergence to a sub-optimal peak). In this study, if ether outcome occurred, models were re-run several times with different initial values for the model parameters.

## Results

### Simulation study 1: False positives under a strictly neutral model with DT substitutions

Recent work suggests that the simplified assumptions employed by models M1a and M2a (e.g., prohibiting DT changes between codons) could negatively impact the inference of positive selection in some cases [18, 20]. To further investigate the impact of DT changes we generated data under the strictly neutral model, with rate 0.06 and 0.03 for DT substitutions respectively. Previous studies found that the false positive rate under the strictly neutral model (without DT substitution) was just 2% for the M1a vs. M2a LRT [11]. By adding DT substitutions, we found that the false positive rate increased to 49% at *α* = 0.05. Imposing additional process-heterogeneity at the DNA level (unequal GTR exchangeabilities and nucleotide frequencies) did not increase the false positive rate (rather, it declined to 22%). The analogous LRTs, carried out under GPP models that exactly match the generating process (G1a^DT^ & G2a^DT^; *α* = 0.05), were much less sensitive. False positives were approximately 4% under equal GTR exchangeabilities and nucleotide frequencies, and when both GTR exchangeabilities and nucleotide frequencies were unequal.

The strictly neutral scenario can be a challenging case for some models because of the large fraction of sites on the boundary of positive selection (50% at *ω* = 1) can make it easy to obtain a false signal for positive selection (*ω*_+_ > 1) by chance at some sites. Indeed, for this reason it is often included in simulation studies as a “benchmark case” (e.g., [7, 11, 45, 46]). The M1a vs. M2a LRT tended to perform well in many previous studies, which did not include DT changes, because the estimates for *ω*_+_ under M2a tended to be only a little > 1 and the estimated proportion of such sites (*p*_+_) tended to be very low. However, by including DT changes in our simulation scenario, the estimates of *ω*_+_ under M2a become upwardly biased in the 5-taxon case (Table 2), which leads to more false positives. To investigate if the relatively long branches in the 5-taxon case represents a worst-case scenario (a large opportunity for DT changes to occur along a single branch), we doubled the number of taxa without increasing the total tree length (case 1c). While the median estimate of *ω*_+_ did get smaller (1.35 in case 1c), the signal for *ω*_+_ > 1 remained significant. This is because estimated value of *p*_+_ increased from 0.28 to 0.49 under M2a when taxon sampling was increased from case 1b (complex model and 5 taxon tree) to case 1c (complex model, 10-taxon tree having shorter branch lengths). The effect of this on the LRT of M1a vs. M2a was an increase in the false positive rate from 22 to 48% (Table 2). Thus, the strategy of sampling additional taxa such that longer branches are shortened does not appear to be effective at mitigating the effect of DT misspecification on the LRT of M1a vs. M2a.

**Table 2:** False positive rates under a strictly neutral evolutionary process with DT nucleotide substitutions between codons

Simulation	LRT false positive rate	median *ω*_+_ and *p*_+_ MLEs		
	M1a - M2a	G1a^DT^ - G2a^DT^	M2a	G2a^DT^
1a (simple, 5 taxa)	0.49	0.04	*ω*_+_ = 6.08	*ω*_+_ = 1.16
			*p*_+_ = 0.37	*p*_+_ = 0.33
1b (complex, 5 taxa)	0.22	0.04	*ω*_+_ = 10.9	*ω*_+_ = 1.37
			*p*_+_ = 0.28	*p*_+_ = 0.20
1c (complex, 10 taxa)	0.48	0.04*	*ω*_+_ = 1.35	*ω*_+_ = 1.02
			*p*_+_ = 0.49	*p*_+_ = 0.35

One hundred replicates (sequence length = 300 codons) were simulated for each scenario. Simulation 1a is based on a simple model (equal DNA exchangeabilities and equal codon frequencies) evolved over a 5-taxon tree. Simulation 1b is based on a more complex generating process using DNA exchangeabilities and codon frequencies derived from a real dataset. Simulation 1b was extended to the case of a 10-taxon tree. Codon models fitted to simulation 1a assumed equal codon frequencies (fequal), and those fitted to simulation 1b used GY94-style F3 × 4 codon frequencies. The asterisk symbol (*) indicates that the results for simulation 1c under the 10-taxon tree is based on 97 replicates due to convergence problems with some datasets

Although these results confirm the suggestion that DT changes can impact the M1a vs. M2a LRT, the strictly neutral scenario is a very unrealistic model for real protein coding sequences. Real sequences will have much more variability among sites in *ω*, and the fraction of strictly neutral sites (i.e., *ω* = 1), if any, will be much less than 50% (e.g., Yang et al. [6]). Moreover, in the case of real data analysis it is extremely unlikely that a fitted model will be an exact match to the true generating process; thus, the impact of model misspecification on the fitted values of *ω*_+_ are unavoidable. For these reasons we explore more realistic evolutionary scenarios in *Simulation Studies 2* and *3*, and we employ both under-fit and over-fit models to carry out the LRTs.

### Simulation study 2: MNRs and more realistic distributions for *ω* variability among sites

Here we explore more realistic scenarios by adding (*i*) greater among-site variability in *ω*, (*ii*) a much smaller fraction of strictly neutral sites, (*iii*) a different GTR process for each position of the codon, and (*iv*) different levels of MNR evolution (Fig. 1b). We withhold DT changes from this study in order to focus on the effect of MNRs (DT changes will be combined with MNR evolution in Simulation Study 3). MNR evolution is induced by using hydrophobicity to determine the relationship between pairs of amino acids and their substitution probability. In this formulation, an H-score of 1 yields an SNR process, whereas an H-score of 0.05 yields a large MNR effect. Note that we do not mean to imply that hydrophobicity is the primary determinant of protein fitness; rather, we use it here as a simple means of inducing unequal exchangeabilities between amino acids. Although far simpler than real data, this MNR-process is sufficient to permit us to explore the impact on parameter estimation and the LRT for positive selection.

Two *ω* distributions without positive selection (Fig. 1b: scenarios 2a and 2b) were employed as a means to investigate false positive rates. Very similar scenarios have been used before for this purpose [11, 45, 46], but assuming a SNR process. Consistent with the results reported in those previous studies, M1a vs M2a (hereafter LRT-1) has low false positives in the SNR case (Table 3: ${e}^{\beta_{HI}}$ = 1). Results were similar for a LRT based on a null GPP model that perfectly fits the data (G1a^*x*^ vs G2a^*x*^: hereafter LRT-2), and a LRT based on a null GPP model that was over-parameterized (G1a^13^ vs G2a^13^: hereafter LRT-3). False positive rates were at, or below, the specified level for all three LRTs even after adding low-MNR and high-MNR to the generating evolutionary process (Table 3: ${e}^{\beta_{HI}}$ = 0.4; ${e}^{\beta_{HI}}$ = 0.05). The only challenge to inference that we observed was a small tendency for convergence problems when using the over-parameterized models in LRT-3. This is not surprising given that the models for LRT-3 are over-parameterized for both number of categories in the *ω* distribution and the amount of MNR. Convergence problems can arise as a consequence of over-parameterization if the likelihood function becomes irregular or discontinuous over the parameter domain [56]. However, the finding that the false positive rate was relatively insensitive to a large MNR effect was surprising given the considerable amount of attention that has been focused on adding MNRs to codon models [17, 18, 20–22, 32].

**Table 3:** False positive rates (null scenarios) and true positive rates (alternative scenarios) for three LRTs when the evolutionary process includes both *ω* variability among sites and MNRs

				SNR (${e}^{\beta_{HI}}$ = 1)			Low MNR (${e}^{\beta_{HI}}$ = 0.4)			High MNR (${e}^{\beta_{HI}}$ = 0.05)		
	*ω*_0_	*ω*_1_	*ω*_2_	LRT-1	LRT-2	LRT-3	LRT-1	LRT-2	LRT-3	LRT-1	LRT-2	LRT-3
*Null scenarios*				*False positives*								
2a	0.05	0.5	1.0	0.00	0.01	0.01^*^	0.00	0.01	0.00^*^	0.00	0.00	0.00^*^
2b		1.0	1.0	0.01	0.04	0.03^*^	0.00	0.03	0.00^*^	0.00	0.03	0.00^*^
*Alternative scenarios*				*True positives*								
2c	0.05	0.5	1.5	0.03	0.36	0.44	0.01	0.24	0.20^*^	0.00	0.09	0.00^*^
2d			2.0	0.52	0.82	0.85	0.05	0.65	0.61^*^	0.00	0.45	0.14^*^
2e			5.0	1.00	1.00	1.00	1.00	0.99	1.00	0.14	0.99	1.00^*^
2f	0.05	1.0	1.5	0.06	0.10	0.08	0.00	0.14	0.05	0.00	0.14	0.01^*^
2 g			2.0	0.33	0.46	0.37	0.00	0.46	0.24	0.00	0.31	0.09^*^
2 h			5.0	1.00	0.99	1.00	0.98	1.00	1.00	0.09	1.00	0.99^*^

LRT-1 compares M1a to M2a (under-fit models). LRT-2 compares G1a^*x*^ to G2a^*x*^ (perfect-fit models). LRT-3 compares G1a^13^ to G2a^13^ (over-fit models). The asterisk symbol (*) indicates scenarios where either convergence problems or suboptimal peaks were encountered for the models of LRT-3. To overcome these problems, models were re-fit to the same dataset multiple times, each using a different set of initial parameter values. The number of problematic datasets for SNR was 2a = 21 and 2b = 1; for low MNR was 2a = 27, 2b = 16, 2c = 16 and 2f = 10; and for high MNR was 2a = 29, 2b = 20, 2c = 35, 2e = 15, 2f = 15 and 2 g = 1. Because using multiple initials for the problematic datasets was successful, the results above are for all 100 replicates

We used scenarios 2c through 2 h to investigate the power of the same three LRTs over a range of signal for positive selection. LRT-based inference about positive selection should get easier with stronger signal for positive selection; i.e., via a bigger gap between *ω*_1_ and *ω*_+_, or with increasing *ω*_+_. This was the case for all three LRTs (Table 3). Power to reject the null was typically larger when there was a bigger gap between *ω*_*1*_ and *ω*_*+*_ (2c-2e vs. 2f-2 h in Table 3) and with increasing values of *ω*_+_ (e.g.*,* 2e > 2d > 2c in Table 3). The LRTs based on the GPP models (LRT-2 & LRT-3) tended to have more power than the traditional test (LRT-1), however all three LRTs performed very well (~ 100%) when the signal is strong enough (2e and 2 h in Table 3). Although the true relationship between these models and any real dataset will be unknown, it is almost certainly the case that the real evolutionary process will be more complex. These results are relevant, as they suggest a tendency for over-simplified models to have less power to detect positive selection.

Next we focused on the impact of MNRs on power by conditioning our comparisons on the signal for positive selection (Table 3: weak = 2c, 2f; moderate = 2d, 2 g; strong = 2e, 2 h). Inducing a low level of MNRs (by setting ${e}^{\beta_{HI}}$ = 0.4) yielded a reduction in power in all LRTs when the signal for positive selection was not strong. The decline was largest for LRT-1 in scenarios 2d (0.53 - > 0.05) and 2 g (0.35 - > 0.00). The effect was similar for LRT-2 and LRT-3 in the same scenarios, but those tests still retained some power (ranging from 0.26 to 0.69). Power was reduced in scenarios 2c and 2f as well. Inducing a high level of MNRs (by setting ${e}^{\beta_{HI}}$ = 0.05) increased the effect. Again, LRT-1 was most affected, as it had substantial reductions in power even in cases where signal for positive selection was strongest (2e and 2 h).

The relationship between the strength of positive selection, the degree of MNR variation, and the power of the LRT is complex. The reason that all methods do best when strong signal for positive selection (*ω*_+_ = 5) is combined with either SNR or low MNRs is because there are more opportunities for nonsynonymous changes having *ω* > 1 to occur along a branch and thereby contribute to the empirical site pattern distributions for those scenarios. Alternatively, when there are high MNRs, nonsynonymous changes having *ω* > 1 occur less frequently, and have less of an influence on the site pattern distribution. For appreciable signal to accumulate in the data, the *ω* must be high (≥5) when there are high MNRs. Furthermore, fitting models M1a and M2a to such data with high MNRs effectively averages the signal over all amino acid differences, regardless of hydrophobicity, thereby yielding reduced estimates for its *ω* values. Hence, the power is very low for LRT-1 (unlike LRT-2 and LRT-3) when there are high MNRs because of two related factors: (*i*) less signal within the site pattern distribution, and (*ii*) lower expected values for the *ω* parameters. Of course, the power of all three tests is negatively impacted by reductions in signal for *ω* > 1, but LRT-2 and LRT-3 were less affected because the GPP models have larger expected values for *ω*. Taken together, the results of *Simulation Study 2* suggest that MNR processes will not necessarily elevate false positive rates; however, true signal for positive selection appears to be harder to detect when a gene has evolved under an MNR process.

### Simulation study 3: Combining DT nucleotide changes between codons with MNRs

This study extends six of the scenarios from *Simulation Study 2* by adding simultaneous DT changes between codons. We chose three distributions for *ω* (one null and two alternative scenarios) and applied both a SNR (${e}^{\beta_{HI}}$ = 1) and a highly variable MNR (${e}^{\beta_{HI}}$ = 0.05) to each. The null scenario in this study (case 3a in Table 4) is more complex as compared to the “benchmark” null (case 1a); this null scenario includes unequal GTR exchangeabilities, a more complex mixture of selective regimes (*ω* distribution) and DT changes. For LRT-1, adding simultaneous DT changes to the more complex SNR case resulted in a false positive rate of 55%. This is consistent with, but larger than, what was observed for LRT-1 in the case 1a employed in *Simulation Study 1* (31%). The false positive rates for LRT-2 and LRT-3 (Table 4), which are based on models that allow DT changes, were below the specified significance level of the LRTs (*α* = 0.05) in the SNR case. Results, however, differed substantially when highly variable MNRs were added to the *null scenario*. The false positive rate for LRT-1 dropped to zero, whereas it was 6% for LRT-2 (perfect fit models) and 10% for LRT-3 (over-fit models).

**Table 4:** False positive rates (null scenarios) and true positive rates (alternative scenarios) for three LRTs when the evolutionary process includes DT nucleotide substitutions between codons, *ω* variability among sites, and MNRs

				SNR (${e}^{\beta_{HI}}$ = 1)			High MNR (${e}^{\beta_{HI}}$ = 0.05)		
	*ω*_0_	*ω*_1_	*ω*_2_	LRT-1	LRT-2	LRT-3	LRT-1	LRT-2	LRT-3
*Null scenarios*				*False positives*					
3a	0.05	1.0	1.0	0.55	0.02	0.03	0.0	0.06^*^	0.10^*^
*Alternative scenarios*				*True positives*					
3b	0.05	0.5	2.0	0.95	0.87	0.92	0.01	0.44	0.26
3c	0.05	1.0	2.0	0.99	0.47	0.46	0.0	0.27	0.18

LRT-1 compares M1a to M2a (under-fit models). LRT-2 compares G1a^*x*^ to G2a^*x*^ (perfect-fit models). LRT-3 compares G1a^13^ to G2a^13^ (over-fit models). The asterisk symbol (*) indicates that the results are based on < 100 replicates due to convergence problems with some datasets when there was high MNRs. For LRT-2 case 3a is based on 99 replicates. LRT-3 case 3a is based on 91 replicates

Interestingly, we experienced convergence problems for some datasets evolved under the *null scenario* with highly variable MNRs. Convergence problems were most frequent for LRT-3, which also had a false positive rate above the specified level of the test. Both phenomena could be related to the over-parameterization of the G2a model of LRT-3. Mingrone et al. [57] recently demonstrated that model M2a employed within LRT-1 could have MLEs with non-standard behaviour in some cases. In their study, instabilities in the parameter estimates arose when the model was over-parameterized relative to low signal for among-site variability in *ω.* As models of LRT-3 are over-parameterized for *both* among-site variability in *ω* and amino acid exchangeability parameters, we may have obtained “irregular estimates” (sensu Mingrone et al. [57]) in case 3a. If there is model irregularity under this setting, then the assumed large sample likelihood theory might not be applicable to LRT-3 in case 3a; this could lead to anti-conservative behaviour (e.g., Mingrone et al. [58]), which is what we observed. It is worth noting that the anti-conservative behaviour of LRT-3 in the high MNR case (10%) was relatively mild in comparison to the anti-conservative behaviour of LRT-1 in the SNR case (55%).

Cases 3b and 3c of this study were used to investigate the combined effect of simultaneous DT nucleotide changes and MNRs on power. As a baseline, power was first assessed for 3b and 3c under the SNR scenario with DT changes. LRT-1 had the highest power in both SNR scenarios. However, since LRT-1 also had a very high false positive rate in SNR case 3a, its power may simply reflect a bias in the direction of the alternative model (M2a) when DT changes are occurring. Such a bias is consistent with the results of *Simulation Study 1* and those reported by Kosiol et al. [18] and De Maio et al. [20]. LRT-2 and LRT-3 had reasonable power (Table 4). As expected, power was lower in case 3c where the gap between *ω*_1_ and *ω*_+_ was the smallest. The addition of MNRs had a dramatic impact on the power of all three LRTs. LRT-1 had almost no power to detect positive selection. Compared to the SNR scenario LRT-2 and LRT-3 had reduced power, with LRT-3 exhibiting the larger decrease of the two.

Taken together, the results of this simulation study suggest that appropriately parameterized G-series models can yield improvements in power over previous LRTs for complex evolutionary scenarios involving both DT changes, and MNRs. However, model complexity requires careful management. LRTs based on too simple a model can lead to excessive false positives in some cases (e.g.*,* LRT-1 in SNR case 3a), whereas naive over-parameterization of the model also has negative consequences (e.g., LRT-3 in MNR cases 3a-3c). In the latter case, failure to meet the regularity conditions otherwise assumed to be in place for likelihood-based inference could have led to MLE instabilities and degraded LRTs. With respect to the problem of meeting regularity conditions, there are several potential solutions for real data. The first is to use nonparametric bootstrapping to screen real data for MLE instabilities (e.g., Baker et al. [15]). However, the computational burden would be very high for complex models such as G2a^13^, making it impractical for large-scale surveys of genes. The second is to develop a method that penalizes unstable mixture weights for *ω* in a way that corrects any bias in the LRT [58, 59]; development of such a method is not trivial and is beyond the scope of this paper. The third is to develop and test parameter selection methods suitable for the GPP models. This also poses a computational burden. Ideally, we need a fast method, perhaps based on carefully chosen heuristics, for finding a good balance between model bias and variance. The problem is that model selection methods that rely on MLEs could be compromised in those cases where there has been a breakdown of the usual regularity conditions [57–59]. New methods for model selection may be warranted.

### Simulation study 4: Performance of alternative formulations of the SNR codon models in the null cases of simulation studies 1–3

We investigated whether an alternative form of either the *ω* distribution, or the parameterization of codon frequencies, could be used within the M-series framework to reduce false positive rates. To investigate the effect of frequency parameterization, we re-analyzed all nine null scenarios with LRT-1 (M1a-M2a) after replacing the F3 × 4 GY frequency parameterization with that of MG (Table 5). The MG parameterization had no effect on false positives in those four cases where the rate had been 0% under GY. In the remaining 5 cases, false positive rates under MG were comparable to, and in some cases much larger than, GY. The lowest non-zero false positive rate was associated with a case with no DT changes between codons [SNR only: case 2b], whereas much higher rates were observed in four other cases where DT changes had occurred [SNR + DT: cases 1a-c, 3a]. This result is not unexpected given that the MG parameterization emphasizes the independence of the mutation process between codon positions, and the process of simultaneous DT change employed to simulate those data is a stronger violation of that independence assumption. It was surprising, however, that the effect was so potent as to yield false positive rates > 90% in two cases. More extensive investigation of the relationship between DT processes and the parameterization of codon frequencies is warranted.

**Table 5:** Sensitivity of false positive rates to the choice of model parameterization under the nine different null scenarios of Simulation Studies 1–3

		SNR + DT cases				SNR (no DT)		High MNR (3a = DT)		
	model	1a	1b	1c	3a	2a	2b	2a	2b	3a
M1a - M2a	GY	0.31	0.22	0.48	0.55	0.0	0.01	0.0	0.0	0.0
M1a - M2a	MG	0.25	0.41	0.91	0.94	0.0	0.15	0.0	0.0	0.0
M8_*ω* = 1_ - M8_*ω* > 1_	GY	0.47	0.24	0.58	0.56	0.0	0.02	0.0	0.0	0.0
M8_*ω* = 1_ - M8_*ω* > 1_	MG	0.44	0.57	0.94	0.97	0.0	0.18	0.0	0.0	0.0

Scenarios 1a and 1b are based on a 5-taxon tree, and 2a, 2b and 3a are based on a 17-taxon tree (see Fig. 1). GY denotes the frequency parameterization of Goldman and Yang [26] where the transition probability is proportional to target codon. MG denotes the frequency parameterization Muse and Gaut [47] where the transition probability is proportional to target nucleotide. Both require frequency estimates for the four nucleotides at each position of the codon (denoted F3 × 4), and thus each requires 9 free parameters

To investigate if an alternative form of the *ω* distribution might help reduce false positive rates within the M-series framework, we re-analyzed all nine null scenarios using a popular alternate LRT that compares M8_*ω* + =1_ to M8_*ω* + ≥ 1_. We applied this LRT under both the MG and GY codon frequency parameterization (Table 5). False positive rates between the two LRTs were generally similar; under the alternate LRT the same four cases had 0 false positives, with the remaining five cases having comparable false positive rates, although slightly higher for M8_*ω* + =1_ vs. M8_*ω* + ≥ 1_. The same relationship between MG and GY was also observed for the alternate LRT; false positive rates were higher under MG, and exceeded 90% in two of the cases. These results are interesting because M8 is based on a discretized *β* distribution, with typically 10 categories used for *ω*. Because this model is far more flexible than the 2 and 3 category *ω* distributions used in M1a and M2a, it is usually viewed as a superior model. Indeed, as measured by likelihood score, M8 will often fit a real dataset much better than either M1a or M2a (e.g., [6, 53]). Nonetheless, our results suggest that the formulation of M8 that yields more power in some scenarios also yields more sensitivity to misspecification in others. We note that greater robustness of the M1a vs. M2a LRT to model misspecification has been suggested previously (e.g., [46]). Taken together, these results support the view that performance depends on a complex relationship between the parameterization of a model and the nature of the signal within a given dataset, and that model performance measured under idealized conditions may not be safely extrapolated to real data having more complex evolutionary dynamics [43, 60].

### Real data analyses

We applied LRT-1 and LRT-3 to a set of 21 real *Streptococcus* sequence alignments. LRT-1 is presumed to represent an under-fit scenario, as it is based on codon models (M1a and M2a) that assume a SNR process and which do not permit DT changes. LRT-1 also represents a typical analysis of real data under the M-series modelling approach as implemented in the CODEML program [54]. LRT-3 is presumed to represent an over-fit scenario, as the models (G1a^13^ and G2a^13^) employ 6 different amino acid properties as a means to model MNRs, and it seems unlikely that all of these are necessary for a given dataset. LRT-3 is based on the default model complexity for the COLD program, so it is used to represent a typical analysis under the G-series modelling approach. The real data results (Table 6) are generally consistent with the simulation results; namely, that LRTs based on the G-series models should have more power, but using over-fit models could lead to convergence problems in some datasets. In our real data analysis, LRT-1 was significant for 1 gene, and marginal in another 3, whereas LRT-3 was significant for 3 genes, and there was only a single marginal case. However, convergence problems were encountered with the G-series models for some genes.

**Table 6:** Results of applying LRT-1 and LRT-3 to the set of 21 real *Streptococcus* sequence alignments

Gene				under-fit models		over-fit models		M2a vs. G2a^13^
	N_C_	N_S_	TL	LRT-1: M1a vs. M2a	M2a MLEs	LRT-3: G1a^13^ vs. G2a^13^	G2a^13^ MLEs	2*Δl*
1	892	19	6.98	N.S.	*ω*_*+*_ = 1.0 *p*_*+*_ = 0.026	N.S.	*ω*_*+*_ = 1.02 *p*_*+*_ = 0	881.3
2	639	16	6.37	N.S.	*ω*_*+*_ = 1.0 *p*_*+*_ = 0.15	*P* < 0.0001	*ω*_*+*_ = 4.9 *p*_*+*_ = 0.028	504.2
3	228	11	3.74	N.S.	*ω*_*+*_ = 1.0 *p*_*+*_ = 0.046	N.S.	*ω*_*+*_ = 1.2 *p*_*+*_ = 0	152.1
4	577	9	8.49	N.S.	*ω*_*+*_ = 1.0 *p*_*+*_ = 0.05	N.S.	*ω*_*+*_ = 1.18 *p*_*+*_ = 0	466.1
5	390	9	5.16	N.S.	*ω*_*+*_ = 1.0 *p*_*+*_ = 0.19	*P* < 0.0001	*ω*_*+*_ = 11.7 *p*_*+*_ = 0.03	109.6
6	348	11	4.5	N.S.	*ω*_*+*_ = 1.0 *p*_*+*_ = 0.04	N.S.	*ω*_*+*_ = 3.11 *p*_*+*_ = 0	113.7
7	184	10	0.37	*P* < 0.0001	*ω*_*+*_ = 5.29 *p*_*+*_ = 0.24	*P* < 0.0001	*ω*_*+*_ = 4.36 *p*_*+*_ = 0.29	71.7
8	169	6	30	N.S.	*ω*_*+*_ = 1.0 *p*_*+*_ = 0.001	N.S.	*ω*_*+*_ = 8.46 *p*_*+*_ = 0.02	130.9
9	227	10	5.46	N.S.	*ω*_*+*_ = 1.0 *p*_*+*_ = 0.25	N.S.	*ω*_*+*_ = 20.5 *p*_*+*_ = 0.14	50.3
10^**†§**^	450	10	2.2	N.S.	*ω*_*+*_ = 1.0 *p*_*+*_ = 0.06	N.S.	*ω*_*+*_ = 1 *p*_*+*_ = 0	14.3
11	444	7	4.6	N.S.	*ω*_*+*_ = 1.0 *p*_*+*_ = 0.31	N.S.	*ω*_*+*_ = 1.03 *p*_*+*_ = 0	109.7
12	473	9	0.45	N.S.	*ω*_*+*_ = 1.0 *p*_*+*_ = 0.21	N.S.	*ω*_*+*_ = 10.6 *p*_*+*_ = 0.007	17.3
13	427	8	0.05	0.10 > *P* > 0.05	*ω*_*+*_ = 15.7 *p*_*+*_ = 0.006	N.S.	*ω*_*+*_ > 99 *p*_*+*_ = 0.02	6.2
14	632	7	0.09	0.10 > *P* > 0.05	*ω*_*+*_ = 15.3 *p*_*+*_ = 0.016	N.S.	*ω*_*+*_ = 22.5 *p*_*+*_ = 0.03	25.1
15^**†**^	209	7	10.3	N.S.	*ω*_*+*_ = 1.0 *p*_*+*_ = 0.05	N.S.	*ω*_*+*_ = 1 *p*_*+*_ = 0	164.5
16	232	6	0.43	0.10 > *P* > 0.05	*ω*_*+*_ = 9.4 *p*_*+*_ = 0.29	N.S.	*ω*_*+*_ = 2.3 *p*_*+*_ = 0.37	49.1
17	661	5	3.3	N.S.	*ω*_*+*_ = 1.0 *p*_*+*_ = 0.27	*P* = 0.051	*ω*_*+*_ = 1.0 *p*_*+*_ = 0.33	220.6
18	564	5	7.7	N.S.	*ω*_*+*_ = 1.0 *p*_*+*_ = 0.5	N.S.	*ω*_*+*_ = 1.3 *p*_*+*_ = 0	171.4
19	261	4	9.5	N.S.	*ω*_*+*_ = 1.0 *p*_*+*_ = 0.04	N.S.	*ω*_*+*_ = 1.0 *p*_*+*_ = 0	113.6
20	201	4	2.2	N.S.	*ω*_*+*_ = 1.0 *p*_*+*_ = 0.03	N.S.	*ω*_*+*_ = 17.8 *p*_*+*_ = 0.04	40.4
21^**†**^	166	4	2.7	N.S.	*ω*_*+*_ = 2.15 *p*_*+*_ = 0.20	N.S.	*ω*_*+*_ = 17.8 *p*_*+*_ = 0.017	34.69

N_C_ is the number of codons in the sequence alignment after removal of sites with ambiguities or indels. N_S_ is the number of gene sequences in the alignment. TL is the total tree length estimated under codon model M0 as the mean number of substitution per codon. N.S. indicates a non-significant LRT. The dagger symbol (**†**) indicates a gene for which likelihood optimization under a G-series model did meet convergence criteria. The two-fold s symbol (§) indicates that the MLEs were obtained by removing tip branches having near-zero lengths and re-fitting the model. The gene names, along with the sequence alignments, are provided in the DRYAD repository [51]

The models utilized by LRT-1 and LRT-3 permit an exploration of the impact of model complexity on the inference of positive selection. The one significant result for LRT-1 (gene 7) does not appear to be a false positive due to DT substitutions, as LRT-3 was also significant for that gene. This is in contrast to the three cases of borderline significance for LRT-1 (genes 13, 14 and 16), where LRT-3 was not significant for any of them. Note that these three borderline cases for LRT-1 occurred in the datasets with the lowest tree lengths. In nearly all of the non-significant cases for LRT-1, the MLEs for M2a indicated either *ω*_+_ ≈ 1 or *p*_+_ ≈ 0. This is expected for M2a when it does not provide a significant improvement over M1a [11, 45, 58]. There was one case (gene 21) where the LRT-1 was not significant and yet both *ω*_+_ and *p*_+_ were large. Exceptionally large estimates for *p*_+_ have been observed for M2a when there is very low signal within the data about the parameters of the *ω* distribution [57]. This was certainly the case for gene 21, which is the shortest gene in the dataset (166 codons) and is represented by just 4 sequences.

In all but three genes (10, 12 and 13), the G-series models yielded very substantial increases in likelihood over the M-series models (Table 6), suggesting that the additional complexity of the G-series models was in many cases warranted. However, because the G-series are likely to be over-fit, we will avoid making direct, or mechanistic, interpretations of the MLEs with respect to the MNR process, or the rate of DT change (see Jones et al. [43, 59] for a discussion of the problem of interpreting confounded parameter estimates). Development and validation of parameter selection methods for the G-series models will ultimately permit us to make inferences about such “background” processes. Nonetheless, our simulation studies indicate that the G-series models, via LRT-3, can be used to make inferences about the impact of positive selection within a gene. Consistent with the expectation for greater power (see Table 4), LRT-3 was highly significant for genes 2, 5 and 7, whereas LRT-1 was significant for one gene. In two of those genes the MLEs for *ω*_+_ and *p*_+_ suggest a small fraction of sites under positive selection (*p*_+_ < 0.03). If those genes were truly evolving under an MNR process, then such low signal would be difficult to detect via LRT-1 (see Simulation Studies 2 and 3).

Signs of G-series convergence problems were observed for three genes (10, 15 and 21). Because LRT-1 and LRT-3 were consistent for genes 15 and 21 (both non-significant), we do not think convergence problems negatively affected the LRTs in those two cases. Convergence problems were more severe for gene 10, but were ameliorated by removing terminal taxa with near zero branch lengths and re-fitting the models to those data. Convergence problems for genes 10, 15 and 21 may be a symptom of over-parameterization of G2a^13^ for those data, which could have led to an irregular likelihood function. A further complication is that the extent to which non-standard behaviours of the MLEs could emerge seems to depend on the details of the true generating process for each gene [57, 58]. In such cases the optimization algorithm can readily produce unreliable parameter estimates (see Mingrone et al. [57] and Suzuki and Nei [61] for empirical examples). For this reason we view the MLEs for these genes with more caution than those obtained from the other genes.

It is important to note that this is not the first report of convergence problems and non-standard MLE behaviours, or of disagreements among model-based LRTs in the analysis of real data. Furthermore, a wide variety of codon models seem to be implicated in such issues. Perhaps the best understood example is the *tax* gene of HTLV. This gene is well known for MLEs that suggest 100% of sites are under positive selection despite having 87% sites being invariant across all 20 lineages that comprise the dataset [61]. Subsequent analyses of the *tax* gene indicate that the implausibly large estimate of sites evolving under positive selection results from violations of statistical regularity conditions [57]. Another example comes from a large-scale survey of primate nuclear receptor genes for spatial and temporal changes in selection pressure [15]. By using a novel method of non-parametric bootstrap (SBA: [57]), they identified non-standard MLE in some nuclear receptor genes and not others [15]. Taking the results of our analysis of 21 real *Streptococcus* genes with those other real data analyses highlights the importance of adopting a standard for best practices that includes a set of reliability and robustness analyses. Bielawski et al. [62] proposed an experimental design, and workflow, that includes a suite of quality control, statistical reliability, and model robustness analyses that can be used to identify problematic datasets under the branch-site style of codon models. We propose that such an “experimental design” should be applied to all computational analyses of real data, regardless of the chosen codon-modelling framework.

## Discussion

We have extended previous work [18, 20] by showing that the LRT based on models M1a and M2a can produce incorrect conclusions about positive selection when both (*i*) nonsynonymous rates depend on the amino acid property and (*ii*) codon substitutions have occurred via DT changes. We have also shown that LRTs can be constructed which have better performance in such scenarios by incorporating additional parameters into the model. However, incorporating too many parameters into a model creates other difficulties, some of which can result in computational problems and inferior performance. More work on model selection methods is clearly warranted. Nonetheless, the over-parameterised models tended to perform better than the under-parameterised models in our simulations, which suggests that there is a role for the G-series models in analyses of real data. We recommend that G-series models should be deployed within a larger experimental design that includes (*i*) assessing robustness of results to model assumptions (e.g., Bielawski et al. [62]), and (*ii*) routine use of the non-parametric bootstrap to assess non-standard behaviour of MLEs (e.g., Mingrone et al. [57]).

Our investigation of M-series models revealed that the choice of *ω* distribution (M2 vs. M8) had a minor impact, whereas the choice of codon frequency parameterization (GY vs. MG) can have a major impact on false positives when DT changes had occurred. While both GY and MG can yield unacceptably high false positives, rates tended to be higher under MG (sometimes exceeding 90%). False positive rates for both the GY and MG style models can be understood using the origin-fixation model framework [63], which is a framework for reconciling population genetic processes with macro-evolutionary dynamics. Origin-fixation models assume that residence times for polymorphisms are much shorter than the time between population mutation events. This yields a macro-evolutionary process that instantaneously “jumps” from one fixed state to another (i.e., codon *i* to *j*) as an embedded Markov chain [63]. Both GY and MG assume that the embedded Markov chain is driven solely by single nucleotide mutations. Thus, both are misspecified if either (*i*) the true mutation process includes simultaneous double or triple changes, or (*ii*) such changes do not occur, but the true process violates the “weak mutation” assumptions of the origin-fixation framework. These two scenarios are unidentifiable within real data by single-change codon models, and either violation (*i*) or (*ii*) could increase false positives. Now consider that case of two codons that differ by 2 or 3 nucleotides over a given branch; for a fixed *ω* value, GY and MG will yield different total probabilities of transition from one end of that branch to the other via a sequence of single nucleotide changes. Thus, when fitting these models to real data, the model that “sees” such a sequence of change as having a lower probability will need to further increase the rate of nonsynonymous substitution (via an increase in *ω*) to explain the evolution of those data. It seems that by emphasizing the independence of the mutation process between codon positions, MG requires even larger values of *ω* to explain rapid evolution between codons that differ by 2 or 3 nucleotides. Models that include parameters for apparent DT changes avoid this effect (e.g., [42, 43] and G1a and G2a used here) regardless of whether the process follows phenomenon (*i*) or (*ii*) above.

There is some subtlety in the interpretation of the nonsynonymous rate when modelling MNRs based on the physiochemical properties of the amino acids. Such models can be interpreted as asserting that there is some degree of evolutionary pressure against changes involving certain amino acid properties. Using hydrophobicity as an example, a large influence on the substitution rate such that ${e}^{\beta_{HI}}=0.05$ means that there is strong selective pressure against changes in hydrophobicity. However, within the constraints of selective pressure against changes in hydrophobicity, there may still exist diversifying selection at some sites, independent of the general tendency to preserve hydrophobicity. This means that there can be natural selection for changes in amino acid which do not affect hydrophobicity, and that the selection against changes to hydrophobicity is reduced at these sites. Thus, hydrophobicity manifests as a phenomenological outcome of several processes, with the nonsynonymous rate reflecting the average tendency towards conservation of hydrophobicity over the entire dataset. When G-series models come to be used to investigate the effect of different aspects of physiochemical constraint in real data (polarity, volume, polar requirement, etc.), we recommend using the methods of Jones et al. [43] to assess the amount of phenomenological load carried by the estimates of parameters that imply physiochemical mechanisms of selection.

The models evaluated here are sometimes referred to as “site models”, as they permit the average intensity of natural selection to vary only over the sites. There is growing interest in using the so-called “branch-site” and “clade-site” mixture models to investigate adaptive protein evolution (e.g.*,* Yang and Nielsen, [8]; Bielawski and Yang, [64]; Zhang et al. [65]; Murrell et al. [66]). Such codon models permit the intensity of selection to vary over branches as well as over sites. Venkat et al. [42] recently demonstrated that false positive rates for the branch-site tests can also be exceptionally high when there are double changes between codons. However it is not yet possible to attribute branch-specific false positives to DT changes in real data, as Jones et al. [43] showed that the DT process and the fundamental process of shifting balance on a fixed fitness landscape are confounded. Both of these non-adaptive processes produce site pattern distributions that are consistent with temporal dynamics in *ω*, with the amount of phenomenological load on *ω* depending on a complex relationship between model and data [43, 60]. While the G-series models can be extended by adding temporal dynamics in *ω* to those models already having DT changes and MNRs, this will likely intensify problems that arise when statistical regularity conditions have not been met [15, 57, 62]. Hence, further work on G-series models should focus on developing and testing new methods for parameter selection. The translation of the G-series models to real data will be better suited by first addressing this important issue.

The issues that we have addressed here (LRT power, LRT accuracy, non-standard MLE behaviour, and convergence problems) reflect different aspects of how the relationship between the model and the data can affect inference, and these issues are relevant to all types of codon models [60]. In this study we have focused on modelling MNRs at the amino acid level, DT changes at the codon level, and the GTR process at the DNA level; however, codon models often make other simplifying assumptions about site independence, reversibility, and homogeneity of the tree topology among sites, to name just a few. While these have been investigated to varying extent (e.g., [67–69]), the traditional ways in which simulation studies have been designed are unable to reveal problems associated with statistical irregularity [56, 57, 60] or reveal the effects of realistic levels of model misspecification [10, 43, 44, 60, 70, 71]. Future development of all codon models, as well as formal assessment of parameter selection methods, will require simulation under much more true-to-life scenarios (e.g., DT changes and various MNR scenarios) and cover greater, and more realistic, levels of model misspecification. Only through such studies are we able to appreciate the kinds of inference issues that we are most likely to encounter in real data, and thereby update our notion of best practices accordingly [60].

## Conclusions

We confirm that failure to model MNRs or DT changes can negatively impact the power and false positive rates of LRTs for positive selection. False positives under codon models M2a and M8 can be very sensitive to DT changes. This is exacerbated by the choice of frequency parameterization (GY vs. MG), with rates sometimes > 90% under MG. The MG parameterization emphasizes the independence of the mutation process between codon positions, and this tends to yield larger fitted values for *ω* when the evolutionary process includes DT changes. We describe a novel modelling-framework, GPP, for codons that allows specification of all possible instantaneous codon substitutions, including MNRs and instantaneous DT nucleotide changes. We note that existing codon models can be specified as special cases of the GPP model. LRTs for positive selection implemented under the GPP framework yield substantial improvements in accuracy and power when the true evolutionary process includes MNRs and DT mutations. But, we also find that over-parameterized models can perform less well, and this can contribute to degraded performance of LRTs. For this reason all codon models (GPP and traditional) should be deployed within an experimental design that includes (*i*) assessing robustness to model assumptions, and (*ii*) investigation of non-standard behaviour of MLEs. Within such a design, GPP models should be used alongside traditional codon models to analyze real data. Further work is needed on methods for parameter selection, especially with regard to their performance under realistic levels of misspecification.

## Additional files

Additional file 1: The GPP model uses a logarithm link function to link the non-zero off-diagonal elements of the 61 × 61 instantaneous rate matrix to a linear model. (PDF 688 kb)

Additional file 2: The 10-taxon tree used in simulation 1c that was obtained by dividing each terminal taxon of the 5-taxon tree (used in 1a and 1b), and then re-distributing the total tree-length evenly among all branches. (PDF 66 kb)

Additional file 3: Specification of hydrophobicity factors in the model, and the matrix of hydrophobicity scores between all amino acids. (PDF 57 kb)

## Abbreviations

DT: Double-triple
GA: Genetic algorithm
GPP: General purpose parametric model
GTR: General time reversible model
GY: Goldman and Yang
HI: Hydrophobicity index
HTLV: Human T-lymphotropic virus
LRT: Likelihood ratio test
MEP: Mixed empirical and parametric model
MG: Muse and Gaut
MLE: Maximum likelihood estimate
MNR: Multiple nonsynonymous rates
PCP: Physiochemical-constrained parametric
REV: Fully reversible model
SBA: Smoothed bootstrap aggregation
SNR: Single nonsynonymous rate
