# Supplementary material for: Improved inference of site-specific positive selection under a generalized parametric codon model when there are multinucleotide mutations and multiple nonsynonymous rates
Source: BMC Evol Biol. 2019 Jan 14;19:22. doi: 10.1186/s12862-018-1326-7 (PMC6332903; doi:10.1186/s12862-018-1326-7)
Supplement: Supplementary file 1 — The GPP model uses a logarithm link function to link the non-zero off-diagonal elements of the 61 × 61 instantaneous rate matrix to a linear model. (PDF 688 kb) [file 12862_2018_1326_MOESM1_ESM.pdf]

Additional file 1

The GPP model uses a logarithm link function to link the non-zero off-diagonal elements of the 61 × 61 instantaneous rate matrix to a linear model format.

The basic idea is to use a link function to link the non-zero parameters in the instantaneous rate matrix to a linear model format — the same idea as used in generalized linear models. We choose to use a logarithm as the link function for the following reasons:

- It is a simple function that maps positive numbers to arbitrary real values, which takes away the constraints on the parameters in estimation.
- The log transformation works best for finding a parametric representation of the empirical codon model rate matrix.
- Existing models use a multiplicative form for elements of the Q matrix, which is converted to our additive framework via the log function.

The R matrix is constructed from the set of parameters $\beta_0, \ldots, \beta_p$, whose values can be any real number, or $-\infty$, as follows:

To each β_i there is a corresponding symmetric 61 × 61 matrix $X^{(i)}$ (in fact, the diagonal elements of this matrix are not important). Now for $j \neq k$, we define r_{jk} by $log(r_{jk}) = \sum_i \beta_i \left(X^{(i)}\right)_{jk}$. The diagonal elements of the R-matrix are then given by the equations $\sum_{j=1}^{61} \pi_r \, \pi_{ij} = 0$ for each i (so $r_{ii} = \frac{\sum_{j \neq i} \pi_j r_{ij}}{\pi_i}$). This parameterization ensures that the off-diagonal π_i elements of the R matrix are non-negative.

The matrices $X^{(i)}$ represent important factors for the instantaneous substitution rates. As an example, if we have only a single parameter β_0 with corresponding matrix $X^{(0)}$ all of whose off-diagonal elements are 1, then our parameterization gives that $r_{ij} = e^{\beta 0}$ for every $i \neq j$. In order to allow the interpretation of branch lengths as expected number of substitutions per site, we require that $\sum_{i=1}^{61} \pi_i \, q_{ii} = -1$. In terms of the R matrix, this says $\sum_{i=1}^{61} \pi_i^2 r_{ii} = 1$, and using the formula for r_{ii} in terms of r_{ij} for $j \neq i$, we get $\sum_{i \neq j} \pi_i \pi_j r_{ij} = 1$. This means that for a single-parameter model, we are obliged to chose β_0 so that $e^{\beta_0} \sum_{i \neq j} \pi_i \pi_j =$

1. That is, $e^{\beta_0} = \frac{1}{1-\sum_{i=1}^{61} \pi_i^2}$. For a parameterization with more parameters, there is still a unique value for β_0 which will give the R matrix the required property. We will therefore always take $X^{(0)}$ to be the matrix all of whose off-diagonal elements are 1, and always chose β_0 so that the scaling requirement for the R matrix is satisfied. When this framework is extended to mixture models, there will be a β_0 coefficient for each component of the mixture, and the scaling restriction on the R matrix will only impose a single linear equation on all these β_0. The effect of this β_0 parameter on the likelihood is exactly the same as multiplying all branch lengths by a constant.
