# Supplementary material for: Improved inference of site-specific positive selection under a generalized parametric codon model when there are multinucleotide mutations and multiple nonsynonymous rates
Source: BMC Evol Biol. 2019 Jan 14;19:22. doi: 10.1186/s12862-018-1326-7 (PMC6332903; doi:10.1186/s12862-018-1326-7)
Supplement: Supplementary file 2 — The 10-taxon tree used in simulation 1c that was obtained by dividing each terminal taxon of the 5-taxon tree (used in 1a and 1b), and then re-distributing the total tree-length evenly among all branches. (PDF 66 kb) [file 12862_2018_1326_MOESM2_ESM.pdf]

Additional file 2

The 10-taxon tree used in simulation 1c obtained by dividing each terminal taxon of the 5-Taxon tree (1a and 1b), and re-distributing the total tree length evenly among all branches.

5-taxon tree used in scenarios 1a and 1b

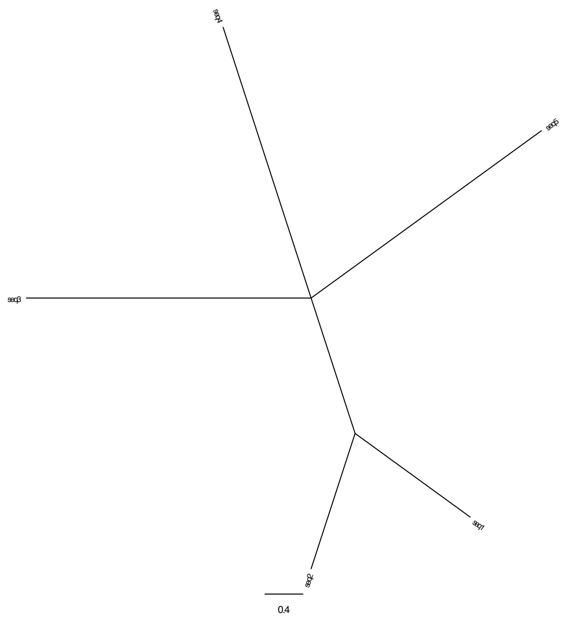

10-taxon tree used in scenarios 1C

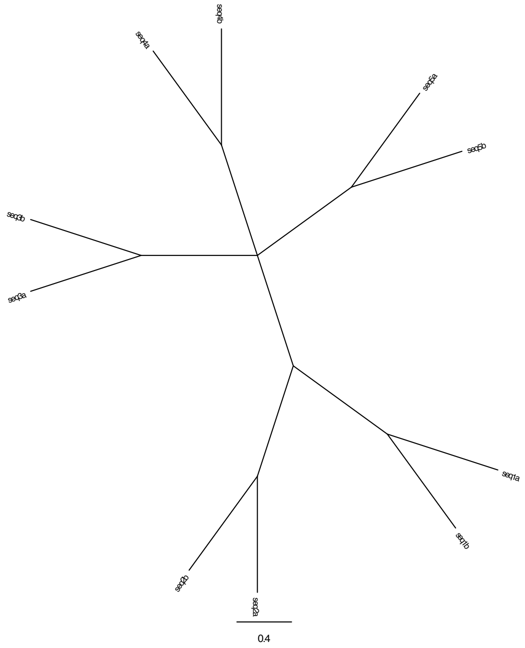

Total tree length = 13.5 substitutions per codon for both trees

5-taxon tree (Simulation Study 1a and 1b):

```
((seq1:1.5, seq2:1.5):1.5, seq3:3, seq4:3, seq5:3);
```

* Note this the tree topology and branch lengths used in the simulation study of Wong et al. (2005) Genetics.168(2):1041-1051.

10-taxon tree (Simulation Study 1c):

```
(((seq1a:0.85,seq1b:0.85):0.85,(seq2a:0.85,seq2b:0.85):0.85):0.85,
(seq3a:0.85,seq3b:0.85):0.85,(seq4a:0.85,seq4b:0.85):0.85,(seq5a:
0.85,seq5b:0.85):0.85);
```
