# Supplementary material for: Improved inference of site-specific positive selection under a generalized parametric codon model when there are multinucleotide mutations and multiple nonsynonymous rates
Source: BMC Evol Biol. 2019 Jan 14;19:22. doi: 10.1186/s12862-018-1326-7 (PMC6332903; doi:10.1186/s12862-018-1326-7)
Supplement: Supplementary file 3 — Specification of hydrophobicity factors in the model, and the matrix of hydrophobicity scores between all amino acids. (PDF 57 kb) [file 12862_2018_1326_MOESM3_ESM.pdf]

Additional file 3

Specifying hydrophobicity factors in the model

The hydrophobicity index of Monera et al. (1995) at pH 7 was re-scaled by a factor of 100, so that Phenylalenine has hydrophobic index 1 and Glycine still has zero. The transformation yields values in the interval [-1,1]. A 20 × 20 pairwise amino acid matrix was then constructed whose entries were the absolute value of the difference between the hydrophobicity scores between all amino acids. This matrix is shown below on the next page.

For the model the exponent of the entries in this matrix were added as a multiplicative factor to the transition rate. That is, if the transition rate from codon i to codon j without the HI parameter is α, and the absolute difference in HI is δ, then the new transition rate is $\alpha e^{\beta\delta}$, where β is the fitted hydrophobicity parameter in the model.

**0.00**	0.08	0.96	0.72	0.59	0.41	0.33	0.58	0.64	0.56	0.33	0.69	0.87	0.51	0.55	0.46	0.28	0.35	0.56	0.22
0.08	**0.00**	1.04	0.80	0.51	0.49	0.41	0.50	0.72	0.48	0.25	0.77	0.95	0.59	0.63	0.54	0.36	0.27	0.48	0.14
0.96	1.04	**0.00**	0.24	1.55	0.55	0.63	1.54	0.32	1.52	1.29	0.27	0.09	0.45	0.41	0.50	0.68	1.31	1.52	1.18
0.72	0.80	0.24	**0.00**	1.31	0.31	0.39	1.30	0.08	1.28	1.05	0.03	0.15	0.21	0.17	0.26	0.44	1.07	1.28	0.94
0.59	0.51	1.55	1.31	**0.00**	1.00	0.92	0.01	1.23	0.03	0.26	1.28	1.46	1.10	1.14	1.05	0.87	0.24	0.03	0.37
0.41	0.49	0.55	0.31	1.00	**0.00**	0.08	0.99	0.23	0.97	0.74	0.28	0.46	0.10	0.14	0.05	0.13	0.76	0.97	0.63
0.33	0.41	0.63	0.39	0.92	0.08	**0.00**	0.91	0.31	0.89	0.66	0.36	0.54	0.18	0.22	0.13	0.05	0.68	0.89	0.55
0.58	0.50	1.54	1.30	0.01	0.99	0.91	**0.00**	1.22	0.02	0.25	1.27	1.45	1.09	1.13	1.04	0.86	0.23	0.02	0.36
0.64	0.72	0.32	0.08	1.23	0.23	0.31	1.22	**0.00**	1.20	0.97	0.05	0.23	0.13	0.09	0.18	0.36	0.99	1.20	0.86
0.56	0.48	1.52	1.28	0.03	0.97	0.89	0.02	1.02	**0.00**	0.23	1.25	1.43	1.07	1.11	1.02	0.84	0.21	0.00	0.34
0.33	0.25	1.29	1.05	0.26	0.74	0.66	0.25	0.97	0.23	**0.00**	1.02	1.20	0.84	0.88	0.79	0.61	0.02	0.23	0.11
0.69	0.77	0.27	0.03	1.28	0.28	0.36	1.27	0.05	1.25	1.02	**0.00**	0.18	0.18	0.14	0.23	0.41	1.04	1.25	0.91
0.87	0.95	0.09	0.15	1.46	0.46	0.54	1.45	0.23	1.43	1.20	0.18	**0.00**	0.36	0.32	0.41	0.59	1.22	1.43	1.09
0.51	0.59	0.45	0.21	1.10	0.10	0.18	1.09	0.13	1.07	0.84	0.18	0.36	**0.00**	0.04	0.05	0.23	0.86	1.07	0.73
0.55	0.63	0.41	0.17	1.14	0.14	0.22	1.13	0.09	1.11	0.88	0.14	0.32	0.04	**0.00**	0.09	0.27	0.90	1.11	0.77
0.46	0.54	0.50	0.26	1.05	0.05	0.13	1.04	0.18	1.02	0.79	0.23	0.41	0.05	0.09	**0.00**	0.18	0.81	1.02	0.68
0.28	0.36	0.68	0.44	0.87	0.13	0.05	0.86	0.36	0.84	0.61	0.41	0.59	0.23	0.27	0.18	**0.00**	0.63	0.84	0.50
0.35	0.27	1.31	1.07	0.24	0.76	0.68	0.23	0.99	0.21	0.02	1.04	1.22	0.86	0.90	0.81	0.63	**0.00**	0.21	0.13
0.56	0.48	1.52	1.28	0.03	0.97	0.89	0.02	1.20	0.00	0.23	1.25	1.43	1.07	1.11	1.02	0.84	0.21	**0.00**	0.34
0.22	0.14	1.18	0.94	0.37	0.63	0.55	0.36	0.86	0.34	0.11	0.91	1.09	0.73	0.77	0.68	0.50	0.13	0.34	**0.00**
